# The Contribution of New Areas to the Total Hirsutism Scores in Basrah Hirsute Women

**DOI:** 10.3390/diseases5040032

**Published:** 2017-11-27

**Authors:** Rudha Naser Hussein, Khalil Ismail Al Hamdi, Abbas Ali Mansour

**Affiliations:** 1 CABMS, Najaf Health Directorate, Najaf 61013, Iraq; rudha_2007@yahoo.com; 2 Department of Medicine, Basrah College of Medicine, Basrah 61013, Iraq; khalil_hamdi2003@yahoo.com

**Keywords:** hirsutism, modified Ferriman-Gallwey, hyperandrogenism, PCOS

## Abstract

*Background:* Hirsutism is the presence of excessive growth of terminal hair in a female in the male-like pattern. It is the most common indicator of hyperandrogenism. The primary objective of this study was to evaluate the clinical impact of new androgens sensitive skin area to total body hirsutism score. *Methods:* This was cross-sectional study. Most of the patients in this study group (*n* = 300) were women of reproductive age group (20–39 years) with a mean age of 26.6 ± 7.1 years. They were recruited in Faiha Specialized Diabetes, Endocrine, and Metabolism Center (FDEMC) during the period from August 2016 to the end of August 2017. All complained from hirsutism and were assessed by using modified Ferriman-Gallwey (m-FG) score system by a single examiner. Each patient underwent detailed clinical assessment in addition to transabdominal or transvaginal ultrasonography of the pelvis with endocrinological investigations. *Results:* Comparison of the mean score at different body areas revealed that new androgens sensitive skin areas (sideburn, lower jaw/neck, buttocks/perineum) were comparable to others area of original m-FG score system or higher than at least three area used in the score. The sideburn area was observed to have the highest score among the new androgens sensitive skin areas. *Conclusion:* Evaluating the terminal hair growth in the new three androgen-sensitive skin areas (sideburn, lower jaw/neck, and buttocks/perineum) were clinically useful in assessing hirsutism score with high impact on total score.

## 1. Introduction

Hirsutism is clinically defined as presence of excessive terminal (coarse) hairs in females in a male-like pattern in androgen-dependent areas. Such androgen-dependent areas include the chin, upper lip, chest, breasts, abdomen, back, and anterior thighs [1,2].

Hirsutism can affect between 5% and 10% of women of reproductive age, and is a common presenting complaint in dermatological outpatient department seeking cosmetic reasons [1].

The Ferriman and Gallway score system was first described in 1961 and has been used to determine the degree of hirsutism. Total of 11 different body areas were used to determine the hair growth, including that on the upper lips, the chin, chest, upper back, lower back, upper abdomen lower abdomen, arm, forearm, thigh, and lower leg [3,4]. About 20 years later, this score decreased to nine areas(upper lips, the chin, chest, upper back, lower back, upper abdomen lower abdomen, arm, and thigh), and was called the modified Ferriman-Gallwey (m-FG) score that was proposed by Hatch et al. in 1981 [4].

Subsequent studies showed that the buttocks/perineum, the sideburn, and lower jaw/neck, contributed more to the total hirsutism score than the nine body areas in the m-FG scoring system. Scoring of hair growth in these new body areas have been included in some other scoring system [5,6].

This study aimed to determine the value of the terminal hair in 12 different androgen-sensitive skin areas, including three areas (sideburn, neck/lower jaw, and buttocks/perineum) to total body hirsutism score.

## 2. Patients and Methods

### 2.1. Design and Participants

Participants for this cross-sectional study were Iraqi women. The chief complaints of all women were hirsutism. They have referred to the Faiha Specialized Diabetes, Endocrine, and Metabolism Center (FDEMC) in Basrah (Southern Iraq). Between August 2016 to the end of August 2017, three hundred premenopausal women with a mean age (26.6 ± 7.1) years and about (70.6%) of patients aged 20–39 years at the time of the enrollment. All of the participants were evaluated for the severity of hirsutism using m-FG scoring [7].

At enrollment, all of the women provided written informed consent, and the ethical committee of the University of Basrah approved the research protocol.

Patients were asked to stop the hormonal medication for a least one month and any way of hair removal for one week before the clinic visit. The participants filled in the hirsutism questionnaire and were examined clinically by one examiner with good experience in applying the m-FG score [7,8].

If patients met the inclusion criteria, Hirsutism was then assessed using the mF-G scoring system. Twelve body areas (upper lip, chin, chest, upper and lower abdomen, upper arms, thighs, upper and lower back sideburn, lower jaw/neck, and buttocks/perineum.) In each of these areas, a score of 0 (absence of terminal hairs) through 4 (extensive terminal hair growth) was assigned. Total scores are obtained for each woman. The maximum score is 36. A score of eight or more was considered as hirsutism according to the m-FG scoring system [3]. The hirsutism severity was considered as mild hirsute for the score of 8–16, moderate for 17–25, and severe for >25 [3,9].

Subjects completed a standardized history form related to physician-diagnosed diseases. Information about a subject’s gynecologic history, such as age at menarche, childbearing history, menstrual cycle length, and regularity and the use of medication, was gathered during the examination

Moreover, signs of frank virilization, including malepattern alopecia, clitoromegaly, deepening of voice, and increased muscle mass, were evaluated [10].

#### 2.1.1. Inclusion Criteria

All of the hirsute women with reproductive age years attending the FDEMC if they accept to participate and has first m-FG score ≥8 were included in the study.

#### 2.1.2. Exclusion Criteria

- Premenarchal or postmenopausal patients
- Patients with drugs history that might interfere with the results e.g., anabolic steroid, metoclopramide, methyldopa, phenothiazine
- Patients who received oral contraceptive pills or/and other anti-androgen drugs in previous three months
- Those who failed to report follow-up visit will be excluded from the study
- Patients with m-FG scoring ˂8
- Pregnancy
- Breastfeeding

### 2.2. Physical Evaluation

The clinical evaluation consisted of a full medical history, including: the menarche date, the onset and rapidity of hirsutism, the presence of symptoms, and signs of endocrine disorders or virilizations (male pattern alopecia, clitoromegaly, deepening of the voice), the metabolic disorders, menstrual and reproductive history, and family and drug history.

Abdominal and pelvic exams (for married women) were performed to look for masses and to evaluate clitoral size if necessary.

Body weight and height were measured by stadiometer (SECA−220)^TM^. The Body mass index (BMI) was calculated by a formula of weight (kg) divided by the square of height (m^2^). 

Blood pressure was measured in all, with two reading 5 min apart and average were taken. Hypertension was defined as drug treatment before or new if blood pressure equal or more than 140 mmHg in systole and or diastolic blood pressure of 90 mmHg.

In the present study, polycystic ovary syndrome (PCOS) was diagnosed according to the Rotterdam Consensus criteria (two of three of following: oligo or anovulation, clinical or/and biochemical evidence of hyperandrogenism, and polycystic ovarian changes) [11]. Idiopathic hirsutism was considered in those who have regular cycles, normal androgens concentration, and no identifiable cause of hirsutism [12].

### 2.3. Laboratory Tests

#### 2.3.1. Basal Hormone Measurement

The blood sampling was done in the early follicular phase of the spontaneous or progesterone-induced menstrual cycle (day 3–5) for hormonal and biochemical analysis [13].

#### 2.3.2. Metabolic Profile

Blood glucose was measured for all using cobas c311 analyzer, and prediabetes and diabetes diagnosis was according to the American Diabetes Association Diagnostic Criteria 2016 [14]. 

### 2.4. Imaging

#### 2.4.1. Ovarian Ultrasonography

The same examiner examined all of the hirsute women. Diagnosis of polycystic ovaries morphology was based on visualization of at least ≥12 follicles in each ovary measuring 2–9 mm in diameter, arranged peripherally over the stroma, and/or increased ovarian volume more than 10 mL with a transabdominal at day two-fifth of mensuration [11].

In most instances, women underwent a transabdominal ultrasound of pelvis, while a minority underwent a transvaginal ultrasound.

#### 2.4.2. Computed Tomography (CT) or Magnetic Resonance Imaging (MRI)

Adrenal CT scanning or MRI to evaluate for either ovarian or adrenal sources of androgen production [15]. It was done to look for an adrenal mass if the woman has a markedly elevated serum total testosterone (TT) or for a serum dehydroepiandrosterone sulfate (DHEA-S) concentration >700 µgm/dL [18.9 µmol/L]) [16].

### 2.5. Statistical Analysis

Data of the 300 hirsutism patients were entered and analyzed by IBM SPSS statistics for Windows, Version 24.0 (IBM Corp., Armonk, NY, USA). Data were checked for errors, inconsistency, and outliers using data cleaning methods. All of the variables were tested for normal distribution using histogram and data exploration. Then parametric statistical tests were used in the analysis. 

Descriptive statistics presented as mean, standard deviation (SD) or standard error of the mean (SEM), frequencies and proportions (%). Receiver operating characteristics (ROC) curve test was used to assess the validity of body areas hirsutism scores in prediction of severe hirsutism. The area under the curve (AUC) performed to calculate each area and compared to each other with the help of the MedCalc software (Z) test.

AUC ranged 0 to 1, the higher AUC more than 0.5 indicated a better predicting ability of a test, and the AUC close to one is the best, valid, and accurate test. Using different cut-off, the sensitivity, specificity, and accuracy were calculated for each of the body areas scores for prediction of severe hirsutism.

## 3. Results

The mean age was 26.6 ± 7.1 years (70.6% aged 20–39 years) at the time of the enrollment. The BMI was normal in 59 (19.7%) women, overweight in 84 (28%), and obese women in 157 (52.3%). Hypertension was seen in in 29 women (9.7%), prediabetes in 12 (4%), and type 2 diabetes in 14 (4.7%).

The cause of hirsutism was PCOS in 204 (68%), while 82 (27.3%) patients had idiopathic hirsutism and 14 (4.7%) were having other diagnoses.

The most affected area was the lower abdomen in 98.3%, upper lip in 97.0%, the chin in 88.3%, and the least one is the upper arm in 40.0% and the chest in 44.7%. The higher mean score was found in the lower abdomen (3.67), followed by chin (2.62), thighs (2.6), sideburn (2.43), and upper lip (2.3). The lowest score was reported in upper back upper arm and chest, where the mean scores for these areas were below one. Buttocks/perineum and lower jaw/neck have a mean score of 1.35 and 1.06, respectively (Table 1).

**Figure 1:**
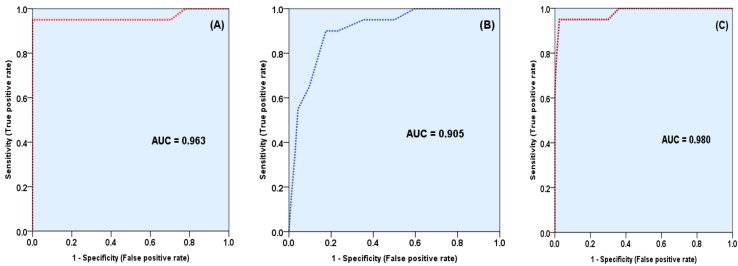
Receiver operating characteristics (ROC) for the validity of total hirsutism score in prediction of severe hirsutism, (**A**) The total score of the nine areas, (**B**) Total score of the three new areas and (**C**) The total score of the 12 areas.

To assess the validity of the three new areas in prediction of severe hirsutism, ROC curve was produced for the total score for the three areas against the severity, the total score of the nine areas (used in m-FG scoring), and the total score for the 12 areas (three new and nine original areas). ROC curve revealed that both scores, of the three and nine areas were significantly good predictor of severe hirsutism, but the total score of nine was stronger than that of three areas, and when both scores used together the score of all the 12 areas, the validity and accuracy was increased and became higher than both score of three or score of nine (Figure 1).

There was no statistically significant difference between a total score of three areas vs. that of nine areas and between total score of nine vs. 12 areas, with *p*-value of 0.5490 and 0.3184, respectively, but the difference was significant when comparing the score of 12 areas vs. that of the three areas, (*p* = 0.0175) (Table 2).

Regarding the area under the curve (AUC) that was produced by with ROC curve for each area (Table 3), it has been found that chest site had the larger AUC (0.938), followed by lower back (0.911), then sideburn (0.904), while lower abdomen showed the least AUC (0.602), preceded by upper lip (0.769). By comparing the AUC of the 12 AUC areas, buttock and perineum area showed significantly higher AUC than upper lip and lower abdomen (*p* < 0.05), and it was comparable to the chin, thigh and upper back (*p* > 0.05 in comparison with these areas). 

AUC of the sideburn area was significantly higher than five of the original nine areas; upper back, thighs, chin, upper lip, and lower abdomen, (in all comparison *p* < 0.05). While AUC of the sideburn area was comparable not significantly different (*p* > 0.05), with each of lower back and upper arm and upper abdomen. AUC of the lower jaw/neck area was comparable to AUC of each upper arm, upper abdomen, upper back thighs, and chin (*p* > 0.05), and was significantly higher than upper lip and lower abdomen.

Among the three androgen-sensitive areas, the sideburn skin area had the highest AUC of 0.904 (CI. 0.883–0.988), higher than the AUC lower jaw/neck were 0.884 (CI. 0.770–0.995), and the latter higher than that of buttock/perineum with AUC 0.815 (CI. 0.627–0.999) (Table 3).

Further analysis using ANOVA to compare the mean hirsutism score of each area across the severity of hirsutism, revealed that in all of the 12 areas the hirsutism score was significantly lower in patients with mild hirsutism and increased with the severity, (*p* < 0.001), and the higher score in all of the areas reported in those patients with severe hirsutism, as it shown in Table 4.

The importance of this comparison to find the relationship between the scores at each of the three new areas with the severity of hirsutism documented by the original mFG score (nine areas). Hence, the significant trend in the change in the total score of the three areas and that for the 12 areas with the severity of hirsutism indicated the direct positive correlation between these scores and severity of hirsutism (Table 4).

## 4. Discussion

In the current study, women with hirsutism were more likely to be obese, a similar finding to that observed in a retrospective study of 161 PCOS women [17].

Although the m-FG scoring system is continuing the most widely used score for assessment of hirsutism existence, it has several limitations, one of these it does not include areas like the sideburns, the lower jaw/neck, and buttock/perineum that is of concern to some women [5,18].

The present cross-sectional study showed that evaluation of terminal hair growth of three new androgen sensitive body areas affected total hirsutism score and its correlation with severity of hirsutism. The sideburn area had the higher impact among these three regions.

These new areas (sideburn area, lower jaw/neck, and buttocks/perineum) showed a higher predictive value, validity, and accuracy in prediction of severe hirsutism, and each area was of higher value than at least two of the other nine areas and comparable to at least three areas, which indicated that the important value of adding these three areas to the total score of 12 areas.

The results of the current study were consistent with a similar finding in Turkey where Hassa et al. performed m-FG scoring system in 12 areas among 65 hirsute women where they also found that the new areas had a significant impact on total hirsutism score [5]. Additionally, in 1995 the new three areas (sideburn area, lower jaw/neck, and buttocks/perineum) was also suggested being included in the scoring system for hirsutism [6].

In the current study, we found that the highest mean score and the main contributor to total body hirsutism score were present in the lower abdomen with the mean score of 3.67. With regarding the new areas, the sideburn area ranked the fourth among the 12 regions, with a mean score of 2.43, which is preceded only by the lower abdomen, the chin, and thigh. The buttocks/perineum in eighth with the mean score of 1.35 and for the lower jaw/neck in ninth score with a mean score of 1.05.

In comparison with Turkish study, their main finding was that the highest score and the main contributor site to total body hirsutism score involved the thigh area with the highest mean score of 2.26 [5,19]. While for new areas it was arranged in different sequences as following: buttocks/perineum ranked in second preceded by thigh with the mean score of 1.83, followed by the sideburn in the third with the mean score of 1.86, and for lower jaw/neck area in ranked the seventh score with the mean score of 1.54, proceeded by the lower abdomen, chin, and thigh [5].

Derksen et al. in one study found that the four areas the upper lip, the chin, lower abdomen, and thigh had highest values, and he also concluded that the sideburn was greater in hirsute than in reference population [18].

At tertiary level institution studies, it has been found that the highest mean score and the main contributor to total body hirsutism score were the chin followed by upper lip, chest, and lower abdomen [20,21].

Another study by Rong li et al. showed that the strongest contributors to hirsutism among different body areas were the lower abdomen, thighs, and upper lip [22].

The second finding of the study was the low impact of areas; like upper back, upper arm, and chest had lower mean score and less contributing effect to total hirsutism score, with a mean score that ranged between 0.72–0.97, which is essentially similar to Hassa et al.’s study, where upper back, upper arm, and upper abdomen had low mean score [5].

The androgen sensitivity of different skin areas differs greatly in the hirsute women, and subsequently, some regions are higher contributors to the overall hirsutism score, while some have a low impact on the total score [23,24].

In a report from the semi quantitative assessment of hirsutism in Dutch study, the least amount of terminal hair were identified on the upper arm, upper back, upper abdomen, and lower abdomen [18].

Additionally, the evaluation of hirsutism of 1034 Turkish women, the least terminal hair was found in the upper back, upper arm, and the lower back [19]. Moreover, due to a low contribution of these areas, they could be ignored, as some suggested [5,22].

Indeed, it has been argued that hirsutism scoring must be population specific and should not be evaluated by a single universal scale, due to the substantial influence of race, ethnicity, and culture on the hirsutism diagnosis [5,25,26]. Results of Turkish study women showed that the buttocks/perineum, sideburn, and neck areas greatly contributed to the total hirsutism score, rather than the upper arm, upper back, and upper abdomen, and the main contributors to hirsutism included thighs, buttoks, sideburns, and lower abdomen [5].

However, in Dutch study that assessment of hair growth on the upper lip, chin, lower abdomen, and thigh is most suitable way to evaluate hirsutism [18].

The third finding in the current study is that the most affected body area was the lower abdomen, the upper lip, and chin, and the least one is the upper arm and the chest.

Hirsutism affects women differently. A variety of regions may be affected in different patients [3]. This is due to the variable sensitivity to serum androgens in the hair follicles of various body regions, and subsequently the response to androgens may be widely different. It is proven that certain skin areas are more sensitive and serve as better indicators of hirsutism [5,27,28].

In comparison with others studies, the affected areas that are of most concerns to the patients include upper lip, chin, chest, and areola [29]. Zhao et al. evaluated twelve locations on the body for hirsutism, they found the lip, chest, and lower abdomen were the main regions for assessing the hirsutism status of women [28].

## 5. Limitation

Despite recruiting a good sample of women, it represents a single Center and it is difficult to extrapolation of these findings to whole Iraqi women, due to regional variability. Transvaginal ultrasound was not performed for all of the participants. Accordingly, some women that were diagnosed as idiopathic hirsutism could be diagnosed as PCOS on ultrasound criteria.

Unfortunately, the present study was unable to provide a specific cut-off value of m-FG hirsutism score because there was no control group used.

## 6. Conclusions

This study suggests that evaluating the terminal hair growth on the new three androgen-sensitive skin areas (sideburns, lower jaw/neck, and buttock/perineum) were comparable to other areas of the original m-FG scoring system, or higher than at least three areas that were used in the original m-FG scoring. Hence, these three new areas were clinically useful in the prediction of hirsutism severity with a high additive value if used with other nine skin areas in the m-FG score, and they are better than upper back, the upper arm, and chest. Therefore, there seems to be a need for a new cutoff point for the diagnosis of hirsutism using the 12 areas scores, instead of the original nine m-FG.

## Figures and Tables

**Table 1:** Mean values and standard errors of hirsutism scores of body areas.

Body Area	Mean	Std. Error	95% Confidence Interval for Mean
Upper lip	2.3	0.12	2.2–2.4
Chin	2.6	0.11	2.5–2.8
Upper back	1.0	0.10	0.9–1.1
Lower back	1.4	0.13	1.2–1.5
Upper arm	0.7	0.04	0.6–0.8
Thigh	2.6	0.18	2.5–2.7
Chest	0.7	0.05	0.6–0.8
Upper abdomen	1.6	0.10	1.5–1.8
Lower abdomen	3.7	0.09	3.6–3.8
Sideburn	2.4	0.16	2.3–2.6
Jaw and neck	1.1	0.04	0.9–1.2
Buttock and perineum	1.4	0.09	1.2–1.5
Total	1.8	0.13	1.7–1.88

***p* Values for multiple comparisons**: Upper lip vs. sideburn = 0.172, chin vs. sideburn = 0.13, thigh vs. sideburn = 0.074 Upper back vs. lower jaw and neck = 0.316 Lower back vs. buttock and perineum 0.69.

**Table 2:** Comparison of area under the curve (AUCs) of the total scores of three areas, nine areas, and 12 areas.

Total Score for Three New Areas vs. Total Score for Nine Areas	
z statistic	0.599
*p* value	*p* = 0.5490
**Total Score for Three New Areas vs. Total for 12 Areas**	
z statistic	2.376
*p* value	*p* = 0.0175
**Total Score for Nine Areas vs. Total Score for 12 Areas**	
z statistic	0.998
*p* value	*p* = 0.3184

**Table 3:** Area under the curve produced by ROC curves of the 12 body areas for prediction of severe hirsutism.

Body Area	Area under the Curve	95% CI	
		Lower Limit	Upper Limit
Chest	0.938	0.879	0.996
Lower back	0.911	0.833	0.988
Sideburn	0.904	0.806	1.000
Upper arm	0.901	0.803	0.997
Upper abdomen	0.898	0.820	0.975
Lower jaw/neck	0.884	0.770	0.995
Upper back	0.879	0.757	0.998
Thighs	0.837	0.700	0.971
Chin	0.829	0.662	0.992
Buttock/perineum	0.815	0.627	0.999
Upper lip	0.769	0.534	0.999
Lower abdomen	0.602	0.406	0.794
Total score for all 12 areas	0.986	0.960	0.999
Total m-FG score 9 areas	0.963	0.934	0.992
Total score for 3 new areas	0.905	0.811	0.997

No statistically significant difference has been found in AUC of the original modified Ferriman-Gallwey (m-FG) of the nine areas and the new three areas.

**Table 4:** Relationship between severity of hirsutism and scores of different body areas and total scores.

Body Areas	Severity of Hirsutism						*p*
	Mild		Moderate		Severe		
	Mean	SE *	Mean	SE	Mean	SE	
Upper lip	1.99	0.07	2.60	0.07	3.10	0.22	<0.001
Chin	2.07	0.11	3.16	0.10	3.95	0.05	<0.001
Upper back	0.53	0.05	1.30	0.09	2.55	0.23	<0.001
Lower back	0.64	0.06	2.08	0.11	3.40	0.17	<0.001
Upper arm	0.25	0.04	1.01	0.10	2.75	0.26	<0.001
Thighs	2.07	0.07	3.13	0.07	3.70	0.11	<0.001
Chest	0.30	0.05	0.96	0.09	2.60	0.21	<0.001
Upper abdomen	1.01	0.07	2.22	0.08	3.25	0.16	<0.001
Lower abdomen	3.48	0.08	3.88	0.04	4.00	0.00	<0.001
Sideburn	1.33	0.07	2.91	0.10	3.90	0.07	<0.001
Lower jaw/neck	0.66	0.08	1.34	0.11	3.00	0.23	<0.001
Buttock/perineum	0.67	0.08	1.92	0.12	2.90	0.27	<0.001
Total m-FG score 9 areas	12.33	0.20	20.34	0.22	28.10	0.47	<0.001
Total score for 3 new areas	3.33	0.21	6.17	0.23	9.40	1.00	<0.001
Total score for all 12 areas	15.67	0.33	26.51	0.40	37.85	1.07	<0.001

* SE: standard error for mean.
